# Fire Proof Schools

**Published:** 1912-04

**Authors:** 


### Fire Proof Schools

THE recent destruction of the Masten Park High School (Buffalo) by fire leads us to ask the development of a sentiment that, hereafter, every school building beyond small one story structures, should be of fire-proof construction. The value of the lives of children and teachers needs no argument. In fact the risk to life in buildings occupied only during the day time, not crowded with highly inflammable material, and with well disciplined occupants, is comparatively small—as instanced in the present case, in which all escaped in spite of the rapidity of the fire.

But the school should be not merely a place for lessons. Gradually there should be accumulated in it, works of art, scientific collections, something more than the ordinary library and laboratory equipment. A valuable conchologic collection was lost in the present fire; only procrastination in placing it, saved another valuable collection.

More than this, a fire proof building is the nearest approach to a clean building. The schools should be fire proof, not only on account of the danger of fire but of infection, not only on account of the immediate dangers of both but as an object lesson, constantly impressed on every pupil, leading to the demand in after life for the same precautionary construction of other buildings.

Finally, in the long run, fire proof buildings are cheaper for the tax-paying public, not only for obvious reasons which will be impressed on the citizens of Buffalo in the next year or two, but because of the indirect economy in fire fighting apparatus.

